# Construction of a chimeric thermoacidophilic beta-endoglucanase

**DOI:** 10.1186/1471-2091-14-11

**Published:** 2013-04-29

**Authors:** Kristina Kufner, Georg Lipps

**Affiliations:** 1 University of Applied Research of Northwestern Switzerland, Gründenstrasse 40, Muttenz, 4132, Switzerland; 2 Biochemistry, University of Bayreuth, Universitätsstrasse 30, Bayreuth, 95447, Germany

## Abstract

**Background:**

The archeaon *Sulfolobus solfataricus* P2 encodes a thermoacidophilic cellulase which shows an extreme acid and thermal stability with a pH optimum at 1.8 and a temperature optimum at 80°C. This extraordinary enzyme could be useful for biotechnological exploitation but the expression and purification in expression hosts like *E*. *coli* is unsatisfactory due to the high aggregation tendency of the recombinant enzyme. The thermophilic cellulase CelA from *Thermotoga maritima* belongs to the same glycoside hydrolase family (GH12) but has a neutral pH optimum. In contrast to SSO1949 this enzyme is expressed partially soluble in *E*. *coli*.

**Results:**

We aimed to constructed a hybrid enzyme based on these two beta-endoglucanases which should successfully combine the advantageous properties of both cellulases, i.e. recombinant expression in *E*. *coli*, acidophily and thermophily. We constructed two hybrid proteins after bioinformatic analysis: both hybrids are expressed insoluble in *E*. *coli*, but one hybrid enzyme was successfully refolded from washed inclusion bodies.

**Conclusions:**

The refolded active chimeric enzyme shows a temperature optimum of approximately 85°C and a pH optimum of approximately pH 3 thus retaining the advantageous properties of the *Sulfolobus* parent enzyme. This study suggests that the targeted construction of chimeric enzymes is an alternative to point mutational engineering efforts as long as parent enzymes with the wanted properties are available.

## Background

Cellulose is the most abundant biopolymer on earth and the main component of plant cell walls. Cellulose makes 35-50% of the dry weight of plants [1] and represents an important alternative source of renewable energy [2]. Cellulose is a linear biopolymer of ß-1,4-glycosidic linked D-glucose molecules. Cellulose molecules usually consist of several thousand glucose units and can form larger crystalline structures via intermolecular hydrogen bonding. For the non-enzymatic hydrolysis of crystalline cellulose high temperatures combined with extreme pH conditions are required [3].

Cellulose can also be hydrolysed under milder conditions by special enzymes called cellulases. Cellulases catalyze the cleavage of ß-1,4-glycosidic bonds in the cellulose. Because of their mode of action and substrate specificity they can be classified into exoglucanases (EC 3.2.1.91), endoglucanases (EC 3.2.1.4) and ß-glucosidases (EC 3.2.1.21) [4]. Exoglucanases split off cellobiose and endoglucanases hydrolyze ß-1,4-glycosidic bonds to decrease the length of the cellulose chains. ß-glucosidases subsequently hydrolyze short oligosaccharides such as cellobiose to glucose [5]. Based on amino acid sequence similarities cellulases may be classified into different GH (glycoside hydrolases) families [4,6]. To date there are 131 GH families; cellulases (E.C. 3.2.1.4) are found in families 5–10, 12, 18, 19, 26, 44, 45, 48, 51, 61, 74 and 124 (http://www.cazy.org/Glycoside-Hydrolases.html). Family 12 comprises endoglucanases from mesophilic and thermophilic archaea, bacteria and fungi.

The demand for stable and highly active cellulases is high [7]. Cellulose as renewable source is an ideal low-cost starting material for the production of bioethanol that can be used as an alternative to fossil fuels. Cellulose in contrast to starch and other agricultural biopolymers has the advantage that it does not compete with the nutritional demands [8]. To make cellulose accessible for enzymatic degradation, the biomass is pre-treated with high temperatures and strong acids. For the next degradation step extreme thermoacidophilic enzymes would be preferable. Most commercial enzymes have a pH optimum near neutrality and are derived from the mesophilic fungus *Trichoderma reesei*. In contrast, the cellulase SSO1949 from the hyperthermophilic archeaon *Sulfolobus solfataricus* represents a thermoacidophilic enzyme, which is optimally adapted to work under acidic conditions and high temperatures.

The enzyme SSO1949 (molecular mass 37 kDa) has a pH-optimum at approximately 1.8 as well as a temperature optimum at approximately 80°C [9]. To our knowledge only the protease thermopsin from *Sulfolobus acidocaldarius*[10] shows a similar pH and temperature activity profile.

The protein consists of a N-terminal signal peptide, a Ser/Thr-rich region and a catalytic domain which shows significant homology to cellulases of glycoside hydrolase family 12 [9]. However, when SSO1949 is expressed in *E*. *coli*, it is mostly insoluble and the preparation of active enzyme through solubilisation and refolding is cumbersome. The inclusion bodies formed by SSO1949 are urea-stable and can only be solubilized with 6 M guanidinium chloride. Refolding is possible by rapid dilution in 0.8 M arginine. However during purification of the refolded enzyme by cation exchange chromatography the protein precipitates on the column and can only be eluted with guanidinium chloride containing buffers. We also attempted to refold and purify SSO1949 at acidic, neutral and alkaline pH values. However the best results were obtained at neutral pH. The high aggregation tendency of SSO1949 precludes its use for commercial applications or further engineering studies.

In the present study, we report the construction of hybrid proteins of the cellulases SSO1949 from *Sulfolobus solfataricus* and CelA from the thermophilic bacterium *Thermotoga maritima* by *in vitro* recombination. *In vitro* recombination allows the combination and optimization of specific properties of different proteins. Ideally, the resulting protein combines the advantageous properties of the parent proteins. Recombination plays a key role in natural evolution of proteins and in the development of antibodies, synthases and proteases [11]. CelA also belongs to GH family 12 and is expressed in our hands in a partially soluble form in *E*. *coli*, but shows a neutral pH optimum [12,13]. We used the program SCHEMA developed by the group of F. Arnold, in order to choose suitable boundaries for chimera construction [14]. Usually, the program SCHEMA is used to construct recombination libraries. Here we show that a more targeted approach with two selected parent enzymes is feasible by obtaining a chimeric enzyme with advantageous properties.

## Results

### Sequence analysis, expression and purification of the hybrid proteins

**Figure 1:**
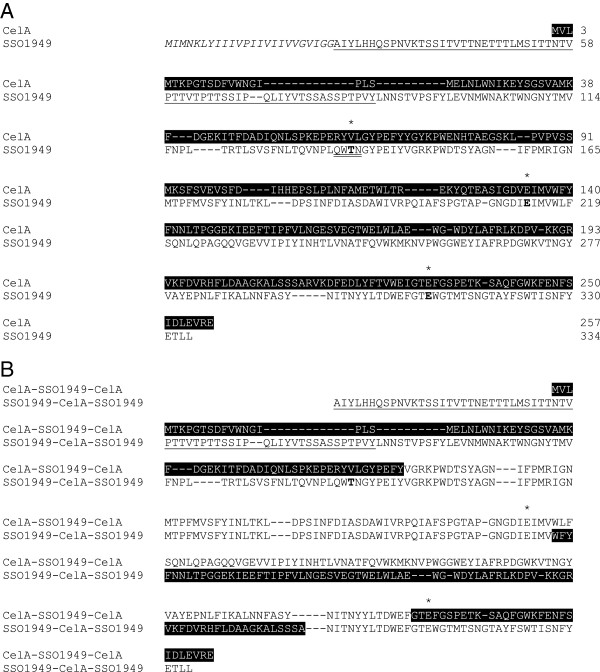
**Alignment of the cellulases SSO1949 from *S. solfataricus *and CelA from *T. maritima. *A**: Alignment of SSO1949 (black letters on white background) and CelA (white on black). The signal peptide of SSO1949 is in italics and the Ser/Thr-rich region is underlined. The conserved catalytic glutamic residues and valine 63 are marked with asterisks. The four amino acids mutated at the valine 63 position are double-underlined. **B**: Sequence of the both fusion proteins. Black letters are amino acids derived from the parental sequence of SSO1949; white letters show residues derived from CelA.

The cellulases SSO1949 from *Sulfolobus solfataricus* and CelA from *Thermotoga maritima* show sequence similarities and belong to GH family 12 (Figure 1). SSO1949 has a temperature and pH optimum of approximately 80°C and approximately pH 1.8 whereas CelA shows maximum activity at approximately 90–95°C and neutral pH.

**Figure 2:**
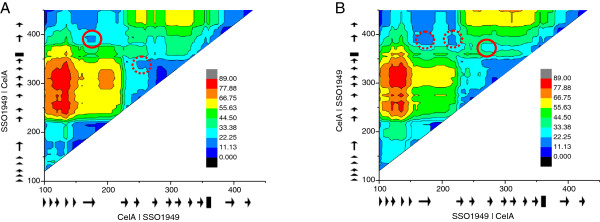
**Calculation of disruption energies for chimeric enzymes.** The disruption energies were calculated in dependence of the two recombination sites for a chimeric protein with the structure protein A - protein B - protein A. The first recombination site is on the abscissa the second on the ordinate; the calculated disruption energies are visualized as a heat map. **A**: energies for the chimera CelA-SSO1949-CelA. **B**: energies for the chimera SSO1949-CelA-SSO1949. The triangles and boxes along the axes represent beta-sheets and alpha-helices, respectively. Red circles refer to the recombination sites selected. Abcissa and ordinate refer to alignment position. The conserved domain of GH12 starts at about alignment position 100.

For construction of the hybrid proteins the program SCHEMA was used. SCHEMA predicts favorable sites for *in vitro* recombination based on structural information [14]. We adapted the python scripts of SCHEMA in order to calculate the disruption energies of a fusion SSO1949-CelA and CelA-SSO1949. This analysis yielded two local minima for the disruption energy at alignment position 175 and 220. However these constructs would have consisted mainly of one parent protein with the N-terminal part of about 100 amino acids substituted by the other parent protein. We have therefore not considered these predictions further. We then calculated the disruption energies for hybrid proteins of the structure CelA-SSO1949-CelA and SSO1949-CelA-SSO1949. The heat maps of the disruption energy as a function of the both recombination sites is shown for both cases in Figure 2. In these triangular shaped heat plots the diagonal represents the case where the middle protein fragment has a length of 20 alignment positions. Likewise proteins corresponding to areas close to the left or upper border contain a very short N-terminal or C-terminal fragment, respectively. The further away from the three borders the more equally distributed are the lengths of the three protein fragments.

**Figure 3:**
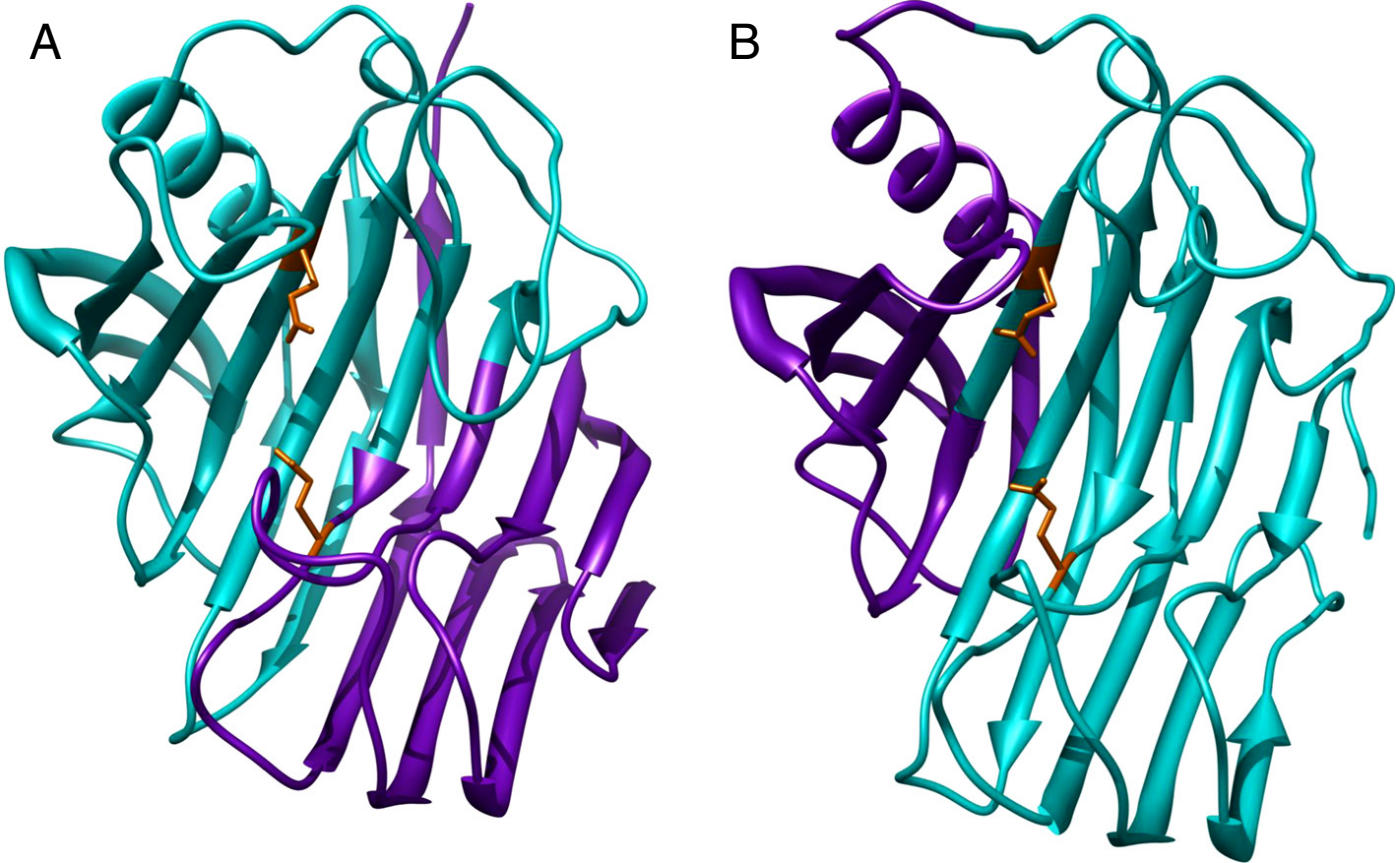
**Model of chimeric enzyme CelA|SSO1949|CelA.** A model structure of the hybrid CelA-SSO1949-CelA (**A**) and of the hybrid SSO1949-CelA-SSO1949 (**B**) using the structure of CelA (3AMH) as template. The sequence alignment was performed with HHpred [15] and the structure calculation was performed with Modeller [16]. The part derived from the parent enzyme CelA is in purple; whereas the SSO1949 part is shown in cyan. The catalytic glutamate residues are shown in orange sticks. It can be clearly seen that the chimeric enzymes consists of two distinct halves derived from both parent proteins. The structures are visualised with Chimera [17].

For the fusion protein CelA-SSO1949-CelA we found low disruption energy for a protein fusion with the first recombination site at alignment position 166 and the second at alignment position 391 (predicted disruption energy 16, red circle in Figure 2A). This leads to a fusion protein of 70 amino acids CelA followed by 163 amino acids SSO1949 and 29 amino acids CelA (Figure 1). We considered this fusion as promising as the center fragment including most of the active center is derived from SSO1949 whose enzymatic properties should be retained in the hybrid enzyme. In the modeled chimeric protein the substrate cleft for the cellulose chain is from the right to the left and is lined by a curved ß-sheet (Figure 3). Indeed the majority of the substrate cleft and the active site are derived from SSO1949 and only the substrate cleft for the non-reducing end stems from CelA. A low disruption energy is also found for a chimeric protein with the recombination sites at positions 260 and 340 (red dotted circle). However in this hybrid enzyme the parent enzyme SSO1949 would make only a minor contribution of about 60 amino acids to the center fragment. We therefore did not consider this possible chimeric enzyme with low disruption energy further.

For the chimeric protein with the structure SSO1949-CelA-Sso1949 we found three possible recombination combinations (see red circles in Figure 2B). The left-most red broken circle would represent the counterpart of the chimera we have chosen for chimera CelA-SSO1949-CelA (Figure 3A). We did not consider this possible chimeric enzyme as the center fragment including most of the active site would be derived from CelA which does not show thermoacidophilic properties we aim for. For the same reason we also refrained from constructing the chimera with the recombination sites around the alignment positions 210 and 390 (right broken red circle). This chimeric protein would also have a dominant center fragment derived from CelA. For the fusion SSO1949-CelA-SSO1949 we therefore choose the recombination sites at positions 272 and 366 (red circle) yielding a fusion protein of 193 amino acids SSO1949 followed by 76 amino acids CelA and 39 amino acids SSO1949. The predicted SCHEMA disruption energy of this chimeric enzyme is somewhat higher with 28 but the parent SSO1949 makes a more prominent contribution to the chimeric protein than with the latter two possibilities. The modeled structure (Figure 3B) shows that the catalytic center with the two catalytic glutamate residues is derived from SSO1949 whereas the reducing end of the substrate binding cleft comes from CelA.

**Figure 4:**
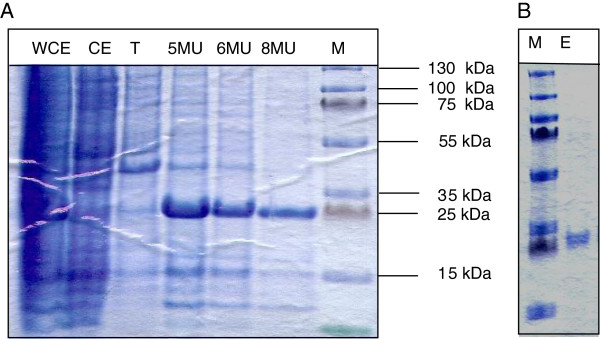
**Purification of recombinant hybrid protein CelA-SSO1949-CelA. (A)** Protein fractions were analysed by SDS-PAGE followed by Coomassie Blue staining: whole cell extract (lane WCE), crude extract (lane CE), Triton X extract (lane T), Urea extract at 5, 6 and 8 M urea, respectively (lanes 5MU, 6MU, 8MU). **(B)** Pooled and dialyzed protein fraction after refolding and hydrophobic interaction chromatography.

Both hybrid proteins were constructed and expressed in *E*. *coli* strain BL21 AI. The proteins were produced in high yields in *E*. *coli* but aggregated in inclusion bodies. The recombinant protein CelA-SSO1949-CelA consists of 262 amino acids and migrates in SDS gels with an apparent mass of approximately 29 kDa, which agrees with the theoretical mass (Figure 4). SSO1949-CelA-SSO1949 consists of 308 amino acids and shows a molecular weight of approximately 36 kDa. The activity of the hybrid proteins was verified by CMC-plates. For this purpose the solubilised inclusion bodies were spotted directly onto carboxymethylcellulose plates. After incubating the plates overnight and staining with Congo Red, only CelA-SSO1949-CelA shows activity. SSO1949-CelA-SSO1949 was inactive in this assay. Noteworthy, both parent enzymes expressed in *E*. *coli* also shows activity in this assay. We therefore concluded that the enzymatic activity of the fusion SSO1949-CelA-SSO1949 is severely compromised and continued working only with the hybrid CelA-SSO1949-CelA.

Because the hybrid protein was insoluble we applied an inclusion body washing step with increasing concentration of urea (Figure 4A) where a majority of the *E*. *coli* host proteins could be removed. Next we refolded the fusion protein. Previously we have screened the refolding of recombinant SSO1949 with 96 buffers and found that 0.8 M arginine containing refolding buffer yield active enzyme (data not shown). Refolding of the fusion protein CelA-SSO1949-CelA with an arginine containing buffer was successful and the soluble and active enzyme was purified and concentrated by hydrophobic interaction chromatography with a propyl-column (Figure 4B).

### Enzymatic characterization of CelA-SSO1949-CelA

For detailed characterization of cellulase activity a FRET (fluorescence resonance energy transfer)-based assay was used. The substrate consists of 6 ß-1,4-linked glucose units and carries a fluorophore (EDANS) at the reducing end and a chromophore at the non-reducing end [18]. Incubation of CelA-SSO1949-CelA with the fluorescent cellohexaoside leads to an increase in fluorescence at 490 nm, which indicates the cleavage of the substrate. The measured fluorescence is proportional to the number of hydrolysed substrate molecules. The FRET-assay is sensitive and the used substrate is even stable under the extreme pH and temperature conditions.

**Figure 5:**
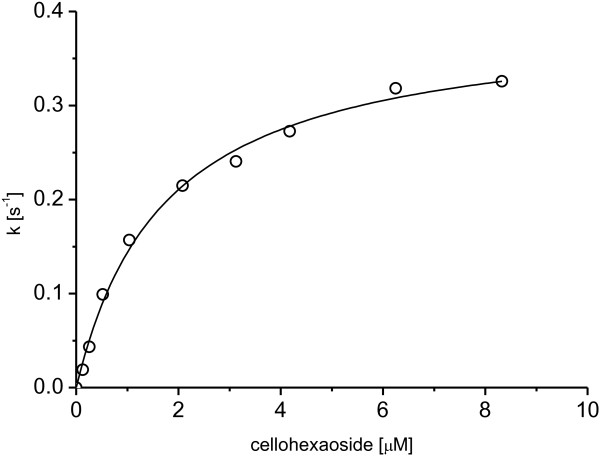
**Michaelis-Menten kinetics of the chimeric enzyme CelA-SSO1949-CelA at pH 3 and 80°C.** The initial rates are shown as a function of fluorescent cellohexaoside concentration. The points are measured rates and the line is the least-squared minimized fit of the data to the Michealis-Menten equation with k_cat_ = 0.39 ± 0.01 s^-1^ and K_M_ = 1.74 ± 0.16 μM.

Measurements of initial rates at various substrate concentrations yielded a K_m_ value of 1.7 μM and the maximal velocity of 0.8 μmol∙min^-1^mg^-1^ at 80°C and pH 3. This value is close to the specific activity of SSO1949 of 1.0 μmole∙min^-1^mg^-1^[9] but much lower than the specific activity of CelA. The maximal velocity of the hybrid protein translates to a k_cat_ of 0.39 s^-1^ under the assumption that the enzyme preparation is wholly active (Figure 5, Table 1). The hybrid protein did not hydrolyse the substrate *p*-nitrophenyl-ß-D-cellobioside. This property of the enzyme has been taken over from SSO1949, which also does not degrade *p*-nitrophenyl-ß-D-cellobioside [9]. Possibly the reducing end substrate binding cleft derived from SSO1949 requires a sugar moiety at the +1 subsite.

**Table 1:** **pH and temperature optimum as well as kinetic parameters of SSO1949**[9]**, CelA and the hybrid protein (this work)**

	**Optimal pH**	**Optimal temperature**	**k**_**cat **_**[s**^**-1**^**]**	**K**_**M **_**[μM]**	**k**_**cat**_**/K**_**M **_**[s**^**-1 **^**μM**^**-1**^**]**
	**80°C1 μM cellohexaoside**	**1 μM cellohexaoside**	**80°C**	**80°C**	**80°C**
**SSO1949**	1.8	80°C	0.5	2.0	0.25
**CelA**	4.5	95°C	19 ± 0.9	5.5 ± 0.8	3.5
**CelA-SSO1949-CelA**	3	85°C	0.39 ± 0.01	1.74 ± 0.15	0.23

### pH and temperature dependence of CelA-SSO1949-CelA

**Figure 6:**
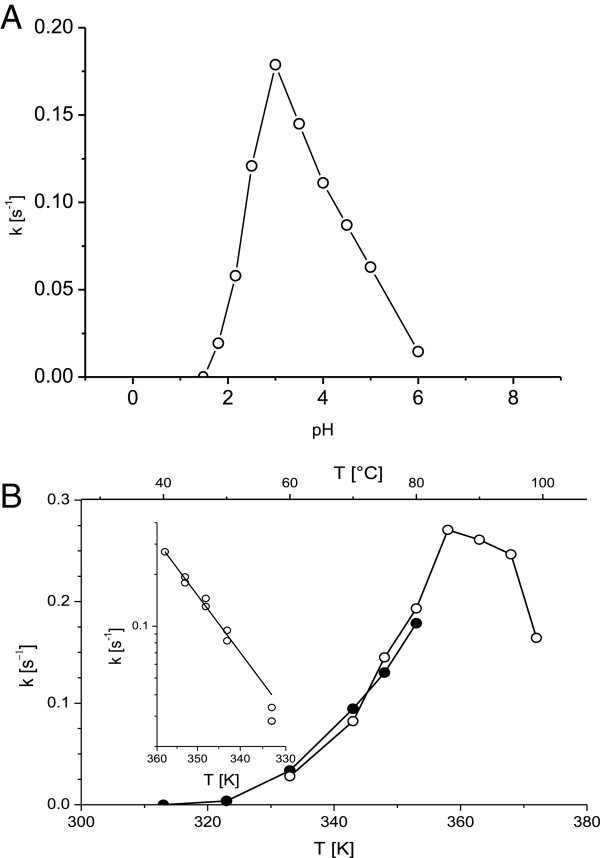
**Activity profile of CelA-SSO1949-CelA at different pH values and temperatures.** (**A**) The cellulase activity of CelA-SSO1949-CelA was assayed at different pH values. The enzyme (120 ng) was incubated with 1 μM fluorescent cellohexaoside in 100 mM phosphate buffer. (**B**) Temperature optimum of the chimeric enzyme in 100 mM sodium phosphate buffer (pH 3): After correcting the temperature dependent fluorescence intensity the activity of the hybrid protein was calculated (●). At temperatures above 80°C the enzymatic hydrolysis as determined with end-point determination after 60 s (о). The inset shows the Arrhenius analysis with reciprocal abscissa and logarithmic ordinate. The enzyme works best at 85°C and pH 3.

The hybrid protein CelA-SSO1949-CelA shows like SSO1949 an optimal activity at acidic pH and high temperatures. The activity measurements with the FRET-substrate at different pH values reveal a pH profile with an optimum at pH 3 (Figure 6). The parents SSO1949 and CelA have a pH optimum at 1.8 for SSO1949 and pH 4.5 for CelA. Endpoint measurements for the hybrid protein revealed a temperature optimum at 85°C. The temperature dependence of the activity allows calculating the activation energy of the enzymatic reaction which is approximately 76 kJ/mol (see inset of Figure 6B). The activation energy for the parent enzyme SSO1949 is 59 kJ/mol [9].

## Discussion

The hyperthermophilic cellulase SSO1949 is optimally adapted to work under acidic conditions and high temperatures. Because of these unique properties, SSO1949 is a good starting point to develop thermoacidophilic cellulases for biotechnological purposes. SSO1949 is expressed insoluble in *E*. *coli* and has a great tendency to aggregate even after refolding therefore large scale production of this protein is difficult to achieve. The thermophilic enzyme CelA in contrast, is expressed in partly soluble form in *E*. *coli*, but shows the highest enzymatic activity at around pH 4.5 for the fluorescent cellohexaoside and pH 6.5 for *p*-nitrophenyl-ß-D-cellobioside (data not shown).

The aim of this study was to develop a hybrid protein in which the three positive characteristics thermophily, acidophily and improved solubility can be combined.

Before constructing the hybrid proteins we used the software SCHEMA to assess putative recombination points for both parent enzymes. The calculations suggested two chimeric proteins, which were then further pursued. CelA-SSO1949-CelA was N- and C-terminally flanked by CelA with the biggest part of the catalytic region of SSO1949. The second protein SSO1949-CelA-SSO1949 consists mainly of SSO1949. Only a part of substrate binding cleft is replaced by CelA. Both hybrid proteins were overexpressed, but in an insoluble form. Furthermore the enzyme SSO1949-CelA-SSO1949 proved to be inactive after refolding attempts. Possibly, in this chimeric protein critical interactions necessary for catalysis have been disturbed. Noteworthy the disruption energy calculated by SCHEMA was also nearly twofold as high as for the other chimeric enzyme.

We were more successful with the hybrid protein CelA-SSO1949-CelA. The protein also aggregated in inclusion bodies but could be refolded into active protein and was far less prone to aggregation than the parent SSO1949. The solubility of the hybrid protein is considerably improved in comparison with SSO1949. The hybrid protein can be completely purified from inclusion bodies using 8 M urea. For purification of SSO1949 stronger denaturants such as guanidinium chloride was needed.

To compare the enzymatic activity of CelA-SSO1949-CelA with SSO1949 and CelA the enzymatic characterization was done with a FRET substrate. The hybrid protein showed a roughly bell-shaped pH profile, which is caused by the ionization states of the two catalytic acidic residues. The pH optimum was approximately at pH 3 which is almost exactly in the middle of the two pH optima of SSO1949 (pH 1.8) and CelA (pH 4.5). By endpoint measurement a temperature optimum of 85°C for CelA-SSO1949-CelA could be determined. The specific activity of the hybrid is lower than the specific activities of the parent proteins. At pH 3 and 80°C the specific activity of CelA-SSO1949-CelA was 0.55 μmole∙min^-1^mg^-1^ with the non-saturating substrate concentration of 1 μM cellohexaoside. The turnover number k_cat_ under the assumption of a fully active enzyme preparation and saturating concentrations of the FRET-substrate is 0.39 s^-1^. This corresponds to a K_cat_/K_M_ value of 2.3 × 10^5^ s^-1^M^-1^.

The molecular basis for the extremely low pH optimum of SSO1949 is currently unknown. There have been a few attempts to influence the pH optimum of endoglucanases by substituting selected amino acid residues in the neighborhood of the catalytic glutamate residues. This approach has been very successful in the case of the Xylanase C from *Aspergillus kawachii*. This enzyme belongs to glycoside hydrolase family 11 which is structurally similar to the glycoside hydrolase family 12. In this enzyme the exchange of an aspartate residue to an asparagine residue raised the pH optimum from 2.0 to 5.0 [19] albeit at the expense of a reduction of the specific activity to only 15%. Similar experiments in the direction to lower the pH optimum of xylanases were much less successful. In the case of the Xylanase A from *Bacillus circulans* the exchange of an asparagine residue to an aspartate residue lowered the pH optimum from 5.7 to 4.6 and increased the specific activity to about 120% [20]. A similar extent of pH optimum change was also seen for the Xylanase I from *Streptomyces sp*. Here the pH optimum dropped from 6.0 to 5.0 when asparagine was exchanged to aspartate at the homologous position. The mutation also resulted in a decrease of the specific activity to about 50%. These studies indicate that this residue in the neighborhood of the catalytic center is important for the pH optimum of the respective enzymes. In an attempt to lower the pH optimum of the *Thermotoga maritima* enzyme CelA we mutated valine 63, the homologous residue. To our disappointment the change V63T using the threonine residue of the *Sulfolobus* enzyme resulted in a mutated enzyme with an unchanged pH optimum but a largely decreased specific activity (− 88%). A second attempt exchanging four amino acids at this position (see Figure 1) resulted in a drop of the pH optimum to 5.5 which is one pH unit lower than the optimal pH with the substrate *p*-Nitrophenyl-beta-D-cellobioside (data not shown). Our failure to achieve a substantial pH change by performing mutations at a selected position known to influence the pH optimum in related enzymes motivated us to construct the chimeric enzymes presented in this work.

## Conclusions

Of the two hybrid enzymes one chimera possess advantageous properties: It still exhibits a low pH optimum, a high temperature optimum and a high specific activity and most importantly it can be easily produced and purified from recombinant *E*. *coli*. This example suggests that the targeted construction of chimeric enzymes is a viable alternative to point mutational studies provided that parent enzymes with the wanted properties are available.

## Methods

### Prediction of chimeric proteins by SCHEMA

The SCHEMA prediction requires a sequence alignment of protein sequences including one structure. The structure is required to calculate the contacts between the residues in the multiple sequence alignment which are then later used to calculate the disruption energies of chimeric enzymes. For the sequence alignment calculated by T-Coffee we used 18 bacterial and archaeal endoglucanase of GH family 12 (ZP_05098279.1, CAB06783.1, YP_001244857.1, YP_002352530.1, ZP_04880023.1, CBH31132.1, NP_578583.1, NP_342800.1, YP_002842958.1, CAB06782.1, YP_001541434.1, YP_001540672.1, YP_921079.1, YP_001541794.1, YP_001540299.1, NP_343873.1, YP_256451.1, YP_002836552.1) which are related to the target enzymes SSO1949 (AAK42142.1) and CelA (CAA93273.1), respectively. The inclusion of the related proteins improved the alignment of the less conserved stretches. As structural template we used the endoglucanase Cel12A from *Rhodothermus marinus* (1H0B) which displayed high sequence similarity to SSO1949 [9]. In contrast to the normal use of SCHEMA we were not interested to design a library of recombination fragments we rather used SCHEMA for a more targeted approach and calculated the disruption energies for the generation of a chimeric protein consisting of SSO1949 and CelA. We took into account two possibilities: a simple fusion of SSO1949 and CelA with a single recombination point and a hybrid protein consisting of three fragments and two recombination points. The python scripts of SCHEMA were adjusted accordingly and calculated the disruption energies for all possible combinations. The calculated disruption energies do not correspond to a physicochemical energy but rather reflects the number of residue contacts which are modified during hybrid construction since one of amino acids which are close in the structure changed their identity. Thus the lower the disruption energy the more native residue contacts could remain preserved in the fusion protein.

### Cloning and expression of the hybrid proteins

For amplification of parts of the genes *sso1949* and *celA* the plasmids pET28c-CelA and pET28c-SSO1949Nhis have been used. The N-terminal part of the hybrid protein CelA-SSO1949-CelA was amplified by PCR with primers celA_BsaI.for (for sequences refer to Table 2) and 1_1949_celA.rev, the middle part with 2_CelA_1949.for and 3_CelA_1949.rev and the C-terminal part with 4_1949_CelA.for and CelA_HindIII.rev. The resulting gene does not encode a purification tag.

**Table 2:** Oligonucleotides used in this study

**Name**	**Sequence (5′ – 3′)**
celA_BsaI.for	AGGTCTCGCATGGTGGTACTGATGACAAAACCGGGAA
celA_HindIII.rev	AAAGCTTTCATTCTCTCACCTCCAGATCAATAGAG
1_1949_celA.rev	CAGGGTTTTCTGCCCACATAAAACTCGGGATAAC
2_CelA_1949.for	GTTATCCCGAGTTTTATGTGGGCAGAAAACCCTG
3_CelA_1949.rev	CTTCCAAACTCG GTTCCGAACTCCCAATCCGTTA
4_1949_CelA.for	TAACGGATTGGGAGTTCGGAACCGAGTTTGGAAG
5_CelA_1949.rev	TTGAAATAGAACCAGACCATTATCTCAATGTCCC
6_1949_CelA.for	GGGACATTGAGATAATGGTCTGGTTCTATTTCAA
7_1949_CelA.rev	TAATAGTTTGTAATGTTAGCAGAACTCGAAAGAG
8_CelA_1949.for	CTCTTTCGAGTTCTGCTAACATTACAAACTATTA
T7-Promotor	TAATACGACTCACTATAGGG
T7-Terminator	GCTAGTTATTGCTCAGCG

SSO1949_CelA_SSO1949 was amplified with primers T7-Promotor and 5_CelA_1949.rev, 6_1949_CelA.for and 7_1949_CelA.rev, 8_CelA_1949.for and T7-Terminator. The resulting gene does no longer encode the signal peptide of SSO1949 but instead encodes an N-terminal hexahistidine peptide and the thrombin recognition sequence.

The generated PCR-fragments contain overlapping ends for *celA* and *sso1949*. In the next step two fragments of each hybrid were amplified together and in the last step the remaining fragment was added and amplified. We created two hybrid genes, which were ligated into the vector pJET/1.2 blunt (Fermentas, St. Leon-Rot, Germany). The hybrid gene celA-sso1949-celA contains cleavage sites *Bsa*I (which cuts outside of its recognition site and generates an *Nco*I compatible end in this construct) and *Hind*III and sso1949-celA-sso1949 contains *Nhe*I and *Xho*I upstream and downstream of the gene. The PCR products were sequenced and cloned into pET-28c (Novagen, Madison, WI, USA).

The expression plasmids were used to transform *Escherichia coli* BL21 AI cells (Invitrogen). For expression, cells were grown overnight in 20 ml of Luria–Bertani medium with 50 *μ*g/ml kanamycin at 37°C. After inoculation of 2 litres of Luria–Bertani medium, the incubation was continued to an A_600_ of 0.6. 1 mM Isopropyl *β*-D-thiogalactoside and 0.2% arabinose were then added and the culture was fermented for further 12 h at room temperature. Cells were harvested by centrifugation.

### Purification and refolding of hybrid proteins

The cell pellet was resuspended in 20 ml in buffer A (100 mM Tris/HCl, pH 7). Cells were disrupted by a sonification and centrifuged for 20 min and 20 000 g. The cell pellet which contains the recombinant enzyme was washed with 20 ml Triton X-100 to remove membrane proteins. The remaining inclusion body was washed with 5 M and 6 M urea and solubilized in 10 ml buffer B (100 mM Tris/HCl pH 8, 8 M urea). After 10 fold dilution in buffer B the protein was refolded in 20 fold volume of refolding buffer (50 mM MES, pH 6, 800 mM arginine) for 2 hours at 8°C. Refolding was done in a total volume of 50 ml. The refolded protein was then purified and concentrated by hydrophobic interaction chromatography. For this purpose the refolded protein solution was first slowly further diluted 4 fold with ddH_2_O and then brought to 1.5 M ammonium sulfate. This solution was loaded onto a 0.3 ml EMD-Propyl (Merck) column (MoBiTec GmbH, Göttingen) and step-eluted with 0.5 ml of 100 mM Tris/HCl pH 8 in 0.5 ml. The active fractions (tested with activity plates) were dialysed against the storage buffer (20 mM sodium phosphate, pH 7.0, 50% (v/v) glycerol, 1 mM DTT, 0.1 mM EDTA, and 150 mM NaCl) and stored at −20°C. The protein concentration was determined spectrophotometrically and calculated according to Ehresmann et al. [21]. Total yield of a 2 litre culture of CelA-SSO1949-CelA was approximately 3 mg of purified protein.

### Activity plates: hydrolysis of carboxymethylcellulose (CMC)

Activity plates enabled qualitative determination of cellulase activity of the chimeric proteins. 2.1 g Gelrite (Sigma) have been autoclaved in 240 ml ddH_2_O and 30 ml of 0.5 M sodium phosphate pH 3, 30 ml of 2% CMC solution and 3 ml of 1 M MgCl_2_ were added and poured into Petri dishes. After solidification 20 μl of protein extract were applied and allowed to dry. The plate was incubated overnight at 75°C. Then, the plate was washed with 100 mM sodium phosphate pH 6 and stained with Congo Red for 30 min. Next, the plate was destained with 1 M NaCl. An active cellulase yields a white spot on a red background.

### Fluorescent activity assay

The quantification of enzyme activity was done by FRET (fluorescence resonance energy transfer) based assay. The bifunctionalized cellohexaoside sodium *N*-{2-*N*-[(S-(4-deoxy-4-dimethylamino-phenylazophenylthioureido-*β*-D-glucopyranosyl)-(1→4)-*β*-D-glucopyranosyl-(1→4)-*β*-D-glucopyranosyl-(1→4)-*β*-D-glucopyranosyl-(1→4)-*β*-D-glucopyranosyl-(1→4)-*β*-D-glucopyranosyl)-2-thioacetyl]aminoethyl}-1-naphthylamine-5-sulphonate was offered as substrate [18]. The reaction was followed by monitoring fluorescence, which increases in the course of the enzymatic reaction. The measurement was performed in 0.1 M sodium phosphate buffer at various pH and temperatures and 1 μM substrate on a PerkinElmer LS50B spectrofluorometer equipped with a thermostatically controlled cuvette holder (80°C). Excitation was at 340 nm and emission was observed at 490 nm. The fluorescent substrate is very stable under these conditions and has a half-live of several hours [9]. Initial rate constants were determined at different substrate concentrations in the presence of 120 ng hybrid protein. The Michaelis–Menten constant *K*_m_, k_cat_ and the maximal velocity v_max_ were calculated by nonlinear regression to the Michealis-Menten equation.

## Competing interest

The authors declare that they have no competing interests.

## Authors’ contributions

KK carried out the cloning, expression, purification and characterization of the enzymes. GL did the SCHEMA analysis and conceived the study. KK and GL wrote the manuscript and approved the final manuscript. Both authors read and approved the final manuscript.
